# Enhancing the interferon-γ release assay through omission of nil and mitogen values

**DOI:** 10.1186/s12931-023-02485-4

**Published:** 2023-07-07

**Authors:** Yun Jung Jung, Ji Eun Park, Ji Won Park, Keu Sung Lee, Wou Young Chung, Joo Hun Park, Seung Soo Sheen, Seulgi You, Joo Sung Sun, Kyung Joo Park, Youn Jung Kim, Kwang Joo Park

**Affiliations:** 1 Department of Pulmonary and Critical Care Medicine, Ajou University School of Medicine, 164 World cup-ro, Suwon, 16499 Gyeonggi-do South Korea; 2 Department of Radiology, Ajou University School of Medicine, Suwon, South Korea; 3 Department of Health and Medical Information, Ajou University Hospital, Suwon, South Korea

**Keywords:** Tuberculosis, Interferon-**γ**, Immunological tests, Latent tuberculosis, Release assay

## Abstract

**Purpose:**

To address the limited utility of the interferon (IFN)-γ release assay (IGRA) caused by its variability and inconsistency.

**Methods:**

This retrospective cohort study was based on data obtained between 2011 and 2019. QuantiFERON-TB Gold-In-Tube was used to measure IFN-γ levels in nil, tuberculosis (TB) antigen, and mitogen tubes.

**Results:**

Of 9,378 cases, 431 had active TB. The non-TB group comprised 1,513 IGRA-positive, 7,202 IGRA-negative, and 232 IGRA-indeterminate cases. Nil-tube IFN-γ levels were significantly higher in the active TB group (median = 0.18 IU/mL; interquartile range: 0.09–0.45 IU/mL) than in the IGRA-positive non-TB (0.11 IU/mL; 0.06–0.23 IU/mL) and IGRA-negative non-TB (0.09 IU/mL; 0.05–0.15 IU/mL) groups (*P* < 0.0001). From receiver operating characteristic analysis, TB antigen tube IFN-γ levels had higher diagnostic utility for active TB than TB antigen minus nil values. In a logistic regression analysis, active TB was the main driver of higher nil values. In the active TB group, after reclassifying the results based on a TB antigen tube IFN-γ level of 0.48 IU/mL, 14/36 cases with negative results and 15/19 cases with indeterminate results became positive, while 1/376 cases with positive results became negative. Overall, the sensitivity for detecting active TB improved from 87.2 to 93.7%.

**Conclusion:**

The results of our comprehensive assessment can aid in IGRA interpretation. Since nil values are governed by TB infection rather than reflecting background noise, TB antigen tube IFN-γ levels should be used without subtracting nil values. Despite indeterminate results, TB antigen tube IFN-γ levels can be informative.

**Supplementary Information:**

The online version contains supplementary material available at 10.1186/s12931-023-02485-4.

## Introduction

Tuberculosis (TB) is a major health concern despite World Health Organization (WHO)-led global control efforts [1]. Latent tuberculosis infection (LTBI) undermines control of TB, while its treatment can prevent development of active TB. However, diagnosis of LTBI is hampered by the lack of a method to identify dormant bacilli; instead, the host immunological response to *Mycobacterium tuberculosis* is evaluated [2].

To diagnose LTBI, the interferon (IFN)-γ release assay (IGRA) has superior performance compared to the tuberculin skin test (TST) [3] However, the multiple procedures and manipulations required for the IGRA lead to variability and inconsistency [4–7], although the utility of the IGRA is difficult to challenge given that LTBI cannot be confirmed using other currently available techniques.

The most widely used IGRA method, QuantiFERON-TB Gold-in-Tube (QFT-GIT), involves TB-antigen-stimulated whole-blood incubation, immunoassay, and subsequent interpretation. The IFN-γ level is measured in incubated blood in a TB-antigen tube (TBAg), a “Nil” tube (Nil; negative control), and a mitogen tube (Mitogen; positive control). To diagnose LTBI, a cutoff value is derived by comparing active TB patients and a population with a very-low likelihood of TB infection, on the premise that IFN-γ levels are identical in active TB and LTBI. The TBAg minus Nil (TBAg − Nil) IFN-γ level is used to eliminate background noise [2, 8].

Studies on blood markers of TB, particularly CXCR3 ligands (also known as IFN-γ-inducible chemokines), have reported several issues with the IGRA [9, 10]. TB-antigen-stimulated marker levels are significantly higher in patients with active TB than in IGRA-positive controls (reflecting LTBI), contradicting the above premise. Furthermore, the chemokine and IFN-γ Nil levels were higher in patients with active TB than in controls, implying that *M. tuberculosis* increases basal levels and subsequent release of markers, even without TB-antigen stimulation. Consequently, subtraction of the Nil IFN-γ level from the TBAg value could compromise the significance of the results. These phenomena were prominently found for chemokines, but were less marked for IFN-γ, which is typically present in samples at low levels. Such issues can lead to false results, particularly near the cutoff values. Furthermore, variability and inconsistency have been reported with regard to indeterminate IGRA results based on abnormally high Nil or low Mitogen levels of IFN-γ [4].

Given these uncertainties and inconsistencies, the IGRA interpretation criteria may have been applied too strictly, leading to inappropriate decision-making and treatment, particularly in countries with high TB prevalence [11, 12]. Therefore, more flexible strategies, such as those based on a zone of uncertainty, have been proposed (but not implemented) [6, 13–16].

To address potential caveats regarding the assay, we analyzed the IGRA results and clinical data from a large population in a university hospital with a relatively high prevalence of TB infection; we focused on actual measured values. Although limited to a single center, this study included prompt and precise processing in a well-controlled laboratory system, which should enhance the reliability of the results [17].

## Methods

### Subjects

We retrospectively evaluated all IGRA tests performed in Ajou University Hospital between November 2011 and July 2019. All valid QFT-GIT results were included in this study. We assessed their purpose and reviewed underlying disease and laboratory data. The data were obtained on the day of blood sampling for the IGRA, but data obtained within 1 week of the IGRA were also used if there had been no change in clinical status.

This study was approved by the Institutional Review Board (AJOUIRB-DB-2022-331) and complied with the Declaration of Helsinki. The requirement for informed consent was waived because this was a very-low-risk retrospective study.

### Diagnostic procedures and criteria

Diagnosis of active pulmonary TB was made according to the WHO guidelines [18]. Bacteriologically diagnosed TB was defined as compatible clinical and bacteriological culture findings or rapid molecular tests, such as polymerase chain reaction (PCR). A clinical diagnosis of TB was defined as compatible clinical, radiological, and pathological findings and an adequate treatment response. The diagnosis of active TB was verified by some of the author-investigators (at least one radiologist and two respiratory medicine specialists).

Diseases were diagnosed according to the clinician’s judgment and the medical records. If necessary, we also referred to the International Classification of Diseases, Tenth Revision [19]. and the available guidelines for individual diseases. Non-tuberculous mycobacteria (NTM) pulmonary infection was diagnosed based on the ATS/IDSA guidelines [20]. Cardiac disease included previous heart attack, heart failure, arrhythmia, ischemic heart diseases, and heart-valve problems requiring maintenance treatment, except for well-controlled hypertension. Immunosuppressive treatment included corticosteroids (≥ 2 mg/kg or ≥ 20 mg of prednisone or equivalent, daily for > 14 days); cytotoxic immunosuppressants for autoimmune diseases, organ transplants, and malignancies; and radiotherapy for malignancies. IGRA positivity was defined according to established criteria [21].

Lymphopenia was defined as a peripheral blood lymphocyte count < 1,000/mm^3^ (or < 2,000/mm^3^ in children < 6 years of age). Neutropenia was defined as an absolute neutrophil count < 2,500/mm^3^, regardless of age. Hypoalbuminemia was defined as a serum albumin level < 3.5 mg/dL. The results of TSTs using RT23 SSI 2TU were included if performed within 1 month of IGRA in cases with no change in clinical status.

### IGRA

Using QFT-GIT (Cellestis, Victoria, Australia; Qiagen, Hilden, Germany) kits, 1 mL of blood was added to each of three Vacutainer tubes precoated with saline (Nil), *M. tuberculosis* ESAT-6, CFP10, and TB 7.7 antigens (TBAg), and phytohemagglutinin (Mitogen). The tubes were incubated for 16–24 h at 37 °C, and plasma was harvested and frozen until further analysis.

IFN-γ levels were measured by enzyme-linked immunosorbent assay according to the manufacturer’s instructions. IFN-γ levels > 10 IU/mL are generally reported simply as > 10 IU/mL, due to the limit of linearity of the standard curve. In this study, the actual values, if available, were used to minimize underestimation of the concentration. However, based on the configuration of the standard curve and the measurements made following serial dilution from the previous study, the actual concentrations were still subject to underestimation because the relevant values were in the non-linear portion of the curve [9].

### Statistical analysis

Data were analyzed using IBM SPSS Statistics for Windows (ver. 25.0; SPSS Inc., Chicago, IL, USA) and MedCalc (ver. 20.115; MedCalc Software, Ostend, Belgium). The data, being nonparametric, are presented as medians with the interquartile range (IQR). Intergroup comparisons were performed using the Mann–Whitney U test and the Kruskal–Wallis test, followed by Bonferroni *post hoc* pairwise comparison. The Fleiss kappa coefficient was used to analyze agreement for categorical data. Spearman’s rank correlation coefficient was used to analyze correlations. Receiver operating characteristic (ROC) analysis was performed to obtain areas under the curve (AUC) and optimal cutoff values according to the Youden index. Logistic regression analyses were performed to identify factors affecting the IGRA results. In all analyses, *P* < 0.05 was considered indicative of statistical significance.

## Results

### Characteristics of the study population

In total, 9,378 cases were included in this study, including 431 in the active TB group. The demographic data are listed in Table 1. The IGRA was performed as part of a general health examination (*n* = 4,307), for diagnosis of LTBI and contact investigation (*n* = 1,624), and to aid in diagnosis or exclusion of active TB (*n* = 3,447). Except for 18 Caucasians, all subjects were Asian.

**Table 1:** Characteristics of the study population

	Active TB	Non-TB		
		IGRA-positive	IGRA-negative	IGRA-indeterminate
No. of cases	431	1,513	7,202	232
Age, years	49 (34–63)	48 (39–57)	31 (24–43)^a^	46 (30–64)
Male, *n* (%)	236 (55.3)	662 (46.0)	2,222 (34.7)	86 (40.0)
Smoking status (*n* = 5,231)	359	1,027	3,630	215
Current	92 (25.6)	198 (19.3)	342 (9.4)	34 (15.8)
Ex-smoker	52 (14.5)	133 (13.0)	244 (6.7)	29 (13.5)
IGRA result				
Positive	376 (87.2)	1,513 (100)		
Negative	36 (8.4)		7,202 (100)	
Indeterminate	19 (4.4)			232 (100)
Close contact to active TB	0 (0)	52 (3.6)	185 (2.8)	1 (0.5)
History of TB	53 (12.5)	148 (10.3)	75 (1.1)^b^	10 (4.7)
NTM infection	7 (1.6)	16 (1.1)	49 (0.8)	3 (1.4)
Corticosteroid use	35 (8.2)	117 (8.1%)	523 (8.0)	105 (48.8)^a^
Other immunosuppressants	12 (2.8)	169 (11.8)	584 (9.0)	58 (27.0)^a^
Underlying conditions				
Diabetes mellitus	48 (11.3)	137 (9.5)	274 (4.2)^a^	29 (13.5)
Autoimmune disease	18 (4.2)	242 (16.8)	749 (11.5)	86 (40.0)^a^
Hematologic malignancy	8 (1.9)	19 (1.3)	58 (0.9)	19 (8.8)^a^
Solid malignancy	14 (3.3)	44 (3.1)	78 (1.2)	13 (6.0)^a^
HIV infection	8 (1.9)	17 (1.2)	112 (1.7)	7 (3.3)
Renal insufficiency	18 (4.2)	36 (2.5)	79 (1.2)	12 (5.6)
Chronic liver disease	3 (0.7)	8 (0.6)	23 (0.4)	6 (2.8)
COPD	10 (2.4)	37 (2.6)	64 (1.0)	9 (4.2)
Cardiac disease	45 (10.6)	112 (7.8)	345 (5.3)	27 (12.6)
Acute infection	9 (2.1)	63 (4.4)	203 (3.1)	46 (21.4)^a^
Laboratory results				
Leukocyte (×10^3^/mm^3^)	6.5 (5.3–8.3)	6.1 (5.0–7.8)	5.9 (4.9–7.5)	8.8 (5.4–13.8)^a^
Lymphocyte (×10^3^/mm^3^)	1.4 (0.9–1.9)	1.8 (1.4–2.3)	1.7 (1.3–2.2)	0.9 (0.5–1.3)^a^
Neutrophil (×10^3^/mm^3^)	4.3 (3.1–5.7)	3.7 (2.8–5.1)	3.6 (2.6–5.1)	7.1 (4.0–11.7)^a^
Albumin (mg/dL)	4.0 (3.3–4.5)	4.4 (3.9–4.7)	4.4 (3.9–4.7)	3.3 (2.8–3.8)^a^
CRP (mg/dL)	3.3 (0.6–6.6)	0.7 (0.1–3.3)	1.0 (0.2–3.9)	4.7 (0.9–12.1)

Data are medians (interquartile range) or *n* (%)

*TB* tuberculosis, non-TB non-tuberculosis, IGRA interferon-γ release assay, NTM non-tuberculous mycobacteria, HIV human immunodeficiency virus, COPD chronic obstructive pulmonary disease, CRP C-reactive protein

^a^*P <* 0.0001 vs. all other groups

^b^*P <* 0.0001 vs. Active TB and IGRA-positive non-TB groups

There were some instances of missing data. History-of-smoking data were obtained in 5,231 cases. Laboratory data were available for C-reactive protein (CRP) in 2,776 cases, for albumin in 2,844 cases, for white blood cell (WBC) counts in 8266 cases, and for differential WBC counts in 5,530 cases.

In the active TB group, 267 had pulmonary TB, 112 had extrapulmonary TB, and 52 had concurrent extrapulmonary-pulmonary TB. *M. tuberculosis* culture was positive in 227 cases, and there were 314 cases of bacteriologically diagnosed TB. The sites of extrapulmonary TB were the pleura (n = 59), intestines (n = 48), lymph nodes (n = 23), peritoneum (n = 14), bones (n = 8), meninges (n = 4), and others (n = 8).

The non-TB group comprised 1,513 IGRA-positive, 7,202 IGRA-negative, and 232 IGRA-indeterminate cases. The IGRA-negative subjects were younger than the IGRA-positive subjects, likely because of the decline in the incidence and prevalence of TB in South Korea [22].

The IGRA-indeterminate non-TB group exhibited a significantly lower lymphocyte count compared with other groups. By contrast, they had higher leukocyte and neutrophil counts. Furthermore, this group showed a significantly higher prevalence of autoimmune diseases, hematologic malignancies, solid malignancies, acute infections, and the use of corticosteroids and immunosuppressants.

After the IGRA, 20 non-TB subjects developed active TB by September 2022; 10 of those cases were IGRA-positive.

### Evaluation of IGRA results

For the intergroup comparison, cases with IGRA-indeterminate results were excluded to ensure validity. The IFN-γ level of TBAg was ≥ 10 IU/mL in 82 cases (18.9%) of active TB and in 132 cases (6.7%) in the IGRA-positive non-TB group. A level of 10 IU/mL was assigned in 20 cases (4.6%) of active TB and 30 (2.0%) IGRA-positive non-TB cases. No Nil values exceeded 10 IU/mL in all cases.

**Fig. 1:**
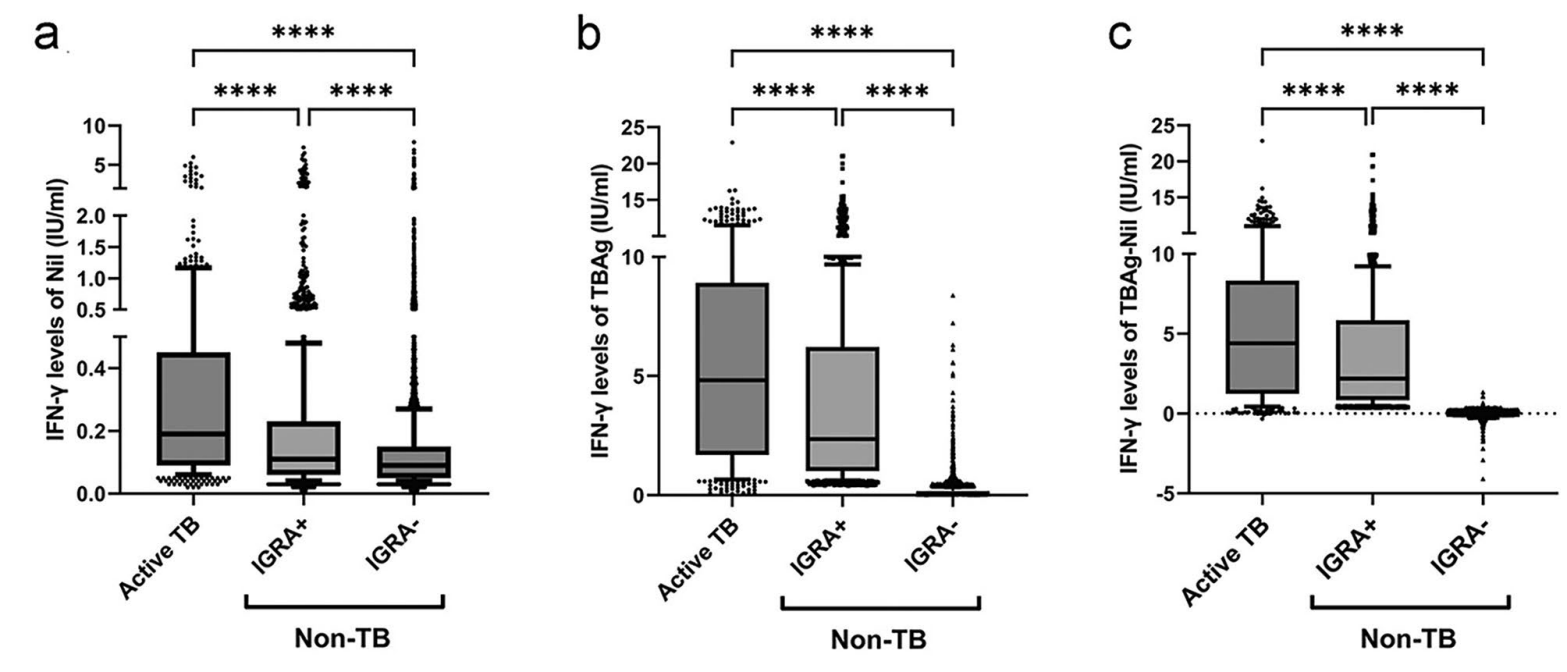
Nil (a), TBAg (b), and TBAg − Nil (c) IFN-γ levels in the active-TB, IGRA-positive non-TB, and IGRA-negative non-TB groups. Cases with indeterminate IGRA results were excluded. Boxes span the 25th to 75th percentiles, whiskers indicate the 10th and 90th percentiles, and bullets represent outliers. *Nil* nil tube, *TBAg* tuberculosis antigen tube, *TBAg − Nil* TBAg minus Nil, *IFN-γ* interferon-γ, *TB* tuberculosis, *IGRA* interferon-γ release assay, *non-TB* non-tuberculosis, *IGRA +* IGRA-positive, *IGRA-* IGRA-negative ****P < 0.0001

The Nil IFN-γ levels were significantly higher in the active TB group (median 0.18 [IQR 0.09–0.45] IU/mL) than in the IGRA-positive non-TB (0.11 [0.06–0.23] IU/mL) and IGRA-negative non-TB (0.09 [0.05–0.15] IU/mL) groups (*P* < 0.0001). The TBAg IFN-γ levels were significantly higher in the active TB group (4.66 [1.51–8.90] IU/mL) than in the IGRA-positive non-TB (2.35 [1.01–6.22] IU/mL) and IGRA-negative non-TB (0.10 [0.06–0.18] IU/mL) groups (*P* < 0.0001). The TBAg − Nil IFN-γ levels were significantly higher in the active TB group (4.07 [1.14–8.17] IU/mL) than in the IGRA-positive non-TB (2.19 [0.84–5.84] IU/mL) and IGRA-negative non-TB (0.01 [− 0.01–0.04] IU/mL) groups (*P* < 0.0001). Additionally, the IGRA-positive non-TB group had significantly higher IFN-γ levels in Nil, TBAg, and TBAg − Nil compared to the IGRA-negative non-TB group (Fig. 1).

**Fig. 2:**
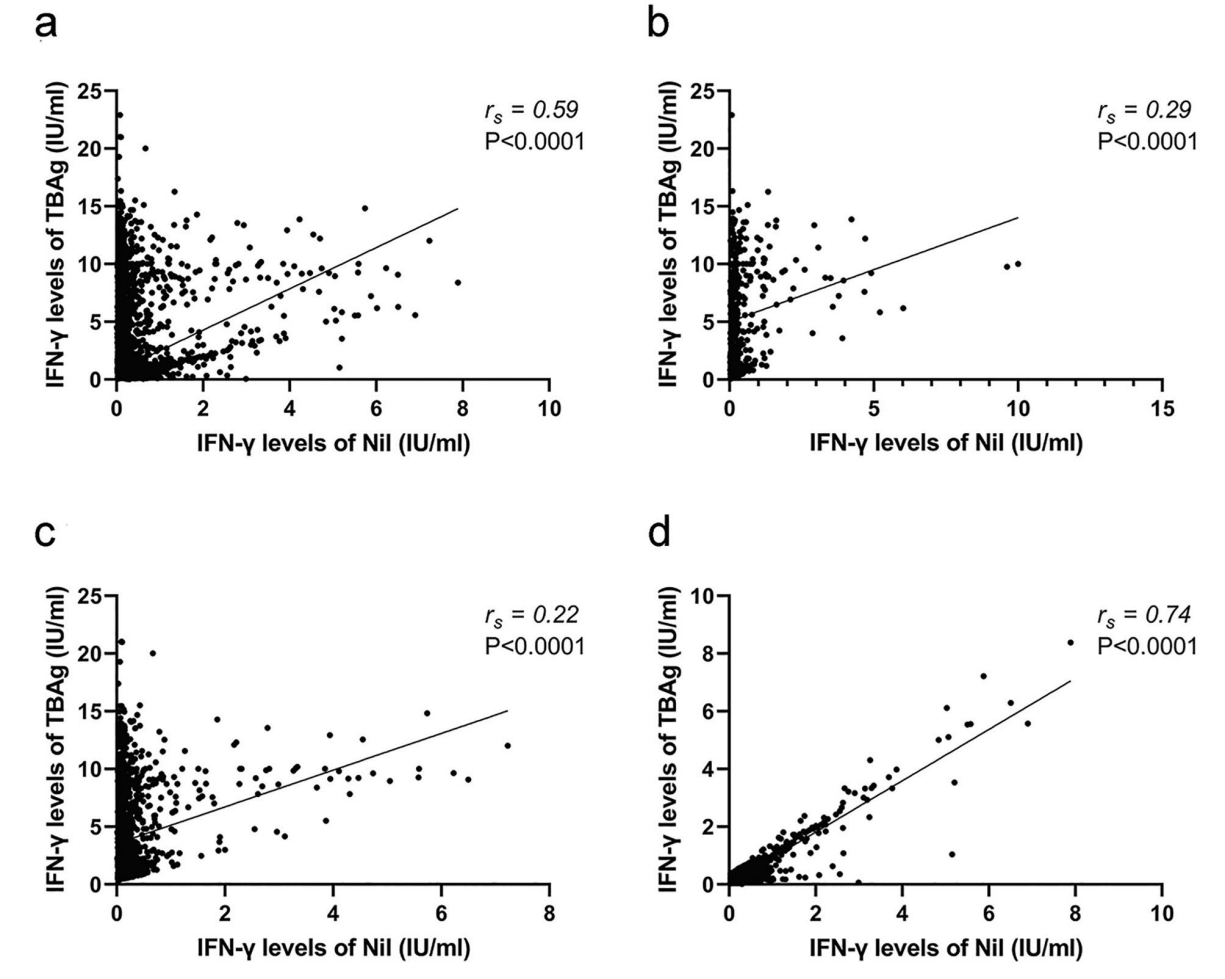
Spearman’s rank correlation analysis of the IFN-γ levels of the Nil and TBAg in all subjects (a) and in the active TB (b), IGRA-positive non-TB (c), and IGRA-negative non-TB (d) groups. *IFN-γ* interferon-γ, *Nil* nil tube, *TBAg* tuberculosis antigen tube, *TB* tuberculosis, *IGRA* interferon-γ release assay, *non-TB* non-tuberculosis

The Nil and TBAg IFN-γ levels correlated significantly in all cases and in the subgroups (Fig. 2). Due to the cases with TBAg IFN-γ levels ≥ 10 IU/mL in the active-TB and IGRA-positive non-TB groups, the correlations may have been underestimated.

With regard to indeterminate results, eight cases had a high Nil IFN-γ level, two of which were active TB cases. Six of these cases were classified as having both high Nil and low Mitogen levels of IFN-γ.

TST was performed in 872 cases, and the agreement between IGRA and TST was significant (κ = 0.46, *P* < 0.001).

### Diagnostic performance

For adequate ROC analysis, combined cases of active TB and LTBI must be differentiated from TB-naïve cases. However, because true LTBI cases could not be identified, active TB cases were differentiated from the non-TB group. ROC analysis revealed that the IFN-γ level of TBAg had a slightly greater AUC than the IFN-γ level of TBAg − Nil in various comparisons (Table 2). The diagnostic performance of TBAg IFN-γ levels was underestimated because both the LTBI cases among the non-TB group and the active TB cases were positively affected by omitting subtraction of the Nil values from the TBAg values. Also, the presence of the LTBI cases resulted in a wide variation of the cutoff value, depending on the different groupings.

**Table 2:** Diagnostic performance of Nil, TBAg, and TBAg–Nil IFN-γ levels for differentiation of active TB cases from those in other groups

	AUC	95% CI	Cut-off	Sensitivity	Specificity
All active-TB cases (n = 412) vs. All non-TB groups (n = 8,715)					
Nil	0.697	0.687 — 0.706	0.19 IU/mL	49.0	80.8
TBAg	0.918	0.912 — 0.923	0.55 IU/mL	93.2	81.6
TBAg − Nil	0.912	0.906 — 0.917	0.38 IU/mL	91.3	83.1
All active-TB cases (n = 412) vs. IGRA-positive non-TB group (n = 1,513)					
Nil	0.623	0.601 — 0.645	0.19 IU/mL	49.0	71.2
TBAg	0.613	0.591 — 0.635	3.38 IU/mL	60.9	59.3
TBAg − Nil	0.597	0.574 — 0.619	3.03 IU/mL	60.0	58.6
Bacteriologically diagnosed active-TB cases (n = 297) vs. All non-TB groups (n = 8,715)					
Nil	0.759	0.751 — 0.768	0.20 IU/mL	58.2	82.3
TBAg	0.921	0.915 — 0.927	0.56 IU/ml	92.9	81.8
TBAg − Nil	0.912	0.907 — 0.918	0.41 IU/mL	90.6	83.6
Bacteriologically diagnosed active-TB cases (n = 297) vs. IGRA-positive Non-TB group (n = 1,513)					
Nil	0.689	0.667 — 0.710	0.19 IU/mL	59.6	71.2
TBAg	0.624	0.602 — 0.647	3.95 IU/mL	59.6	62.7
TBAg − Nil	0.601	0.578 — 0.624	2.47 IU/mL	65.3	54.1

IGRA-indeterminate cases were excluded

*Nil* nil tube, *TBAg* tuberculosis antigen tube, *TBAg − Nil* TBAg tube minus Nil tube, *IFN-γ* interferon-γ, *TB* tuberculosis, *AUC* area under the curve, *CI* confidence interval, *non-TB* non-tuberculosis, *IGRA* interferon-γ release assay

### Factors affecting IGRA results

For binary logistic regression analyses, the subjects were divided into high- and low-IFN-γ level groups according to the median Nil, TBAg, and TBAg − Nil IFN-γ levels (0.09, 0.14, and 0.02 IU/mL, respectively). The groups and IGRA positivity were designated as dependent variables. Underlying and accompanying diseases, and laboratory results, were used as predictors. Predictors that were significant in univariate analyses were entered into the multivariate analysis. For missing data, multiple imputation was performed to maximize the use of all complete data [23].

**Fig. 3:**
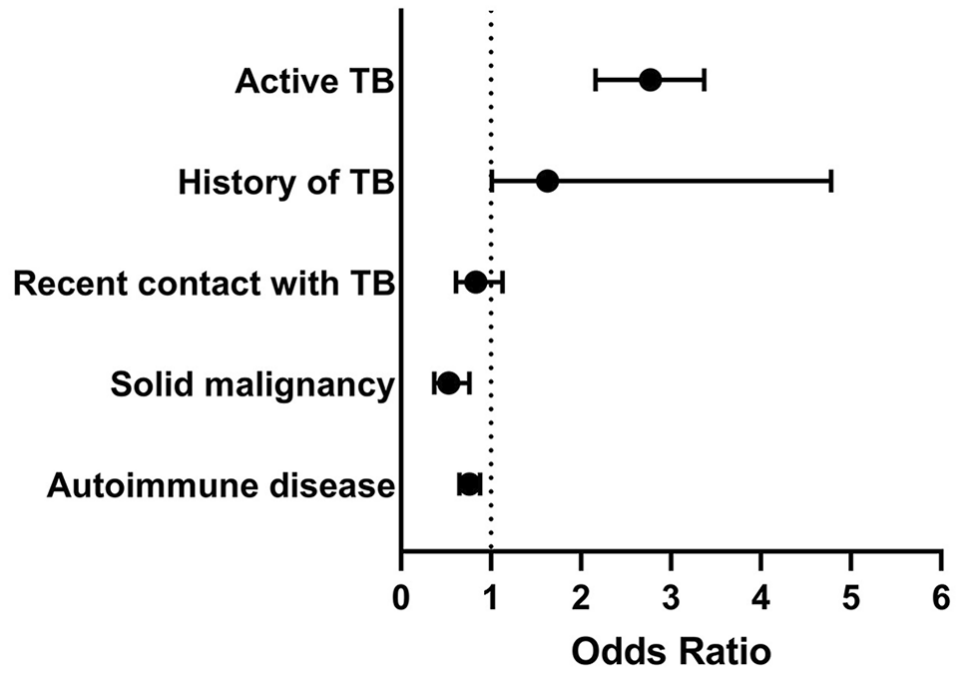
Forest plot of significant factors in the multivariate logistic regression to predict high Nil IFN-γ levels in the IGRA (QFT-GIT). Cases were divided into high- and low-IFN-γ Nil groups based on the median value (0.09 IU/mL). Cases with indeterminate results were excluded. *Nil* nil tube, *IFN-γ* interferon-γ, *IGRA* interferon-γ release assay, *QFT-GIT* QuantiFERON-TB Gold-in-Tube, *TB* tuberculosis

Using a high IFN-γ level of TBAg as the dependent variable, active TB, a history of TB, age, and sex were significant independent predictors (Table S1). Using a high IFN-γ level of Nil as the dependent variable, active TB was the most significant independent predictor. A history of TB, a solid malignancy, and autoimmune diseases showed significant results, but the latter two had negative coefficients probably due to the effects of immunosuppressive treatment. None of the other immunologic, inflammatory, and infectious diseases were identified as significant predictors for high Nil values (Table 3; Fig. 3). Using a high IFN-γ level of TBAg − Nil as the dependent variable, active TB, age, a history of TB, and autoimmune diseases were independent predictors (Table S2). The odds ratio of active TB was higher for the IFN-γ level of TBAg (62.34) than for that of TBAg − Nil (39.09). Using a positive IGRA result as the dependent variable, active TB, a history of TB, age, sex, smoking, NTM infection, and hypoalbuminemia were independent predictors (Table S3). Chronic diseases were collectively evaluated to evaluate their impact on the Nil IFN-γ levels. The Nil IFN-γ levels were not different between groups with and without chronic diseases (Table S4). In addition, when a high IFN-γ level of Nil was used as the dependent variable and chronic disease collectively was used as an independent variable, the presence of chronic disease was not significant, and only active TB and a history of TB were significant independent predictors (Table S5; Fig. S1).

**Table 3:** Univariate and multivariate binary logistic regression analyses to identify factors associated with high Nil IFN-γ levels in the IGRA (QFT-GIT)

Variable	Univariate			Multivariate		
	OR	95% CI	*P* value	OR	95% CI	*P* value
Age, years						
≤ 14	1.23	0.91–1.68	0.183	1.23	0.90–1.68	0.200
15–47	Reference			Reference		
48–63	1.07	0.96–1.19	0.210	1.08	0.96–1.20	0.200
≥ 64	1.18	1.00-1.39	0.049	1.16	0.97–1.37	0.105
Sex (male/female)	0.97	0.89–1.05	0.432			
Smoking status						
Non-smoker	Reference					
Smoker	0.88	0.75–1.05	0.147			
Ex-smoker	0.92	0.76–1.13	0.439			
Active TB	2.87	2.31–3.57	< 0.0001	2.77	2.16–3.37	< 0.0001
History of TB	1.43	1.14–1.81	0.002	1.28	1.01–1.63	0.044
Recent contact with TB	0.75	0.58–0.97	0.031	0.83	0.61–1.13	0.238
NTM infection	1.36	0.86–2.15	0.194			
Hematologic malignancy	1.29	0.86–1.94	0.212			
Renal insufficiency	0.93	0.67–1.30	0.672			
Solid malignancy	0.62	0.44–0.88	0.008	0.53	0.37–0.76	0.001
Diabetes mellitus	0.96	0.80–1.15	0.679			
Chronic liver disease	0.83	0.43–1.59	0.573			
HIV infection	1.11	0.80–1.54	0.548			
Cardiac disease	1.05	0.88–1.24	0.614			
COPD	1.06	0.75–1.49	0.754			
Autoimmune disease	0.71	0.63–0.81	< 0.0001	0.76	0.65–0.88	0.0003
Corticosteroids	0.89	0.77–1.03	0.106			
Immunosuppressant	0.75	0.65–0.86	< 0.0001	0.90	0.77–1.07	0.226
Acute infection	0.89	0.70–1.13	0.329			
Lymphopenia	1.17	1.11–1.23	0.051	1.12	0.96–1.30	0.160
Neutropenia	1.10	0.94–1.29	0.224	1.11	0.95–1.30	0.185
CRP	1.01	0.99–1.03	0.245			
Hypoalbuminemia	1.19	0.98–1.44	0.081			

Cases were divided into high- and low-IFN-γ Nil groups based on the median value (0.09 IU/mL). Cases with indeterminate results were excluded. For definitions of lymphopenia, neutropenia, and hypoalbuminemia, refer to the Methods

*Nil* nil tube, *IFN-γ* interferon-γ, *IGRA* interferon-γ release assay, *QFT-GIT* QuantiFERON-TB Gold-in-Tube, *OR* odds ratio, *CI* confidence interval, *TB* tuberculosis, *NTM* non-tuberculous mycobacteria, *HIV* human immunodeficiency virus, *COPD* chronic obstructive pulmonary disease, *CRP* C-reactive protein

**Fig. 4:**
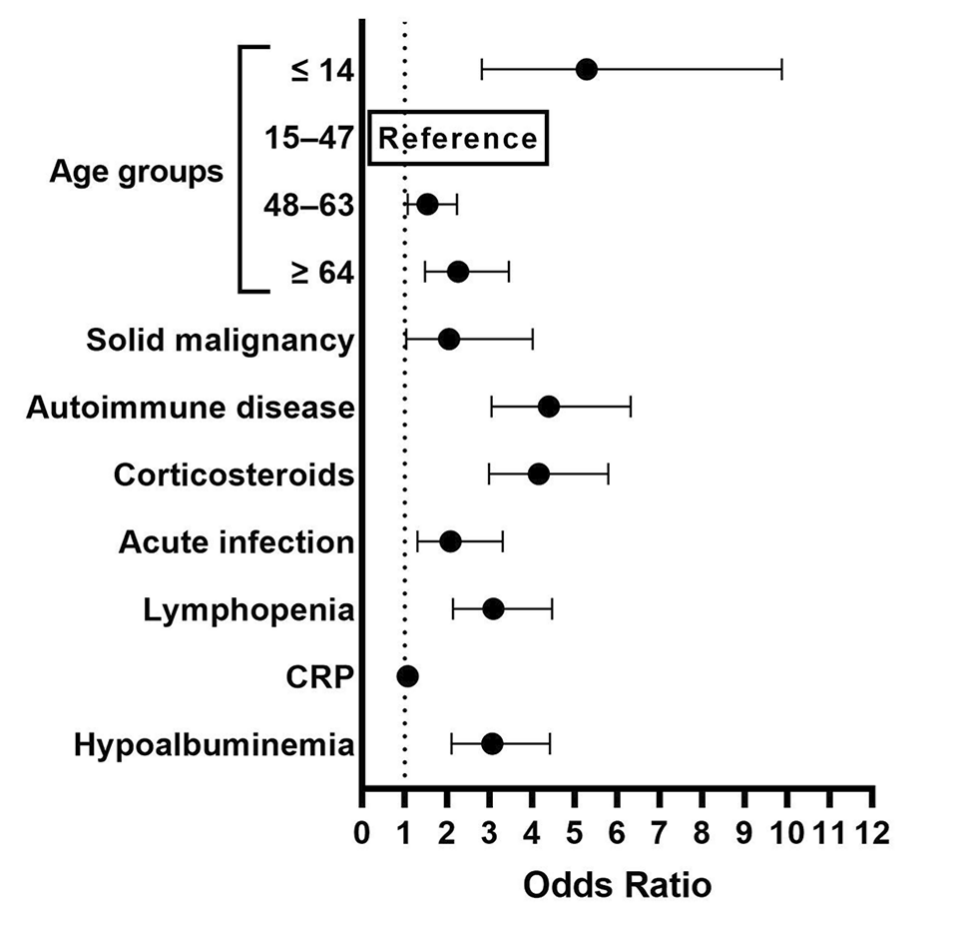
Forest plot of significant factors in the multivariate logistic regression to predict indeterminate results in the IGRA (QFT-GIT). Cases were divided according to the criterion for indeterminate results (Mitogen − Nil IFN-γ level < 0.5 IU/mL). For definitions of lymphopenia and hypoalbuminemia, refer to the Methods. *IGRA* interferon-γ release assay, *QFT-GIT* QuantiFERON-TB Gold-in-Tube, *Mitogen* mitogen tube, *Nil* nil tube, *IFN-γ* interferon-γ

With regard to the indeterminate results, the demographic characteristics of the cases are presented in Table S6. When an indeterminate IGRA result was used as the dependent variable, age, a solid malignancy, autoimmune disease, corticosteroid use, acute infection, lymphopenia, CRP, and hypoalbuminemia were independent predictors (Table 4; Fig. 4).

**Table 4:** Univariate and multivariate binary logistic regression analyses to identify factors associated with indeterminate results due to low Mitogen IFN-γ levels in the IGRA (QFT-GIT)

Variable	Univariate			Multivariate		
	OR	95% CI	*P* value	OR	95% CI	*P* value
Age groups						
≤ 14	7.52	6.10–9.27	< 0.0001	5.28	2.82–9.88	< 0.0001
15–47	Reference			Reference		
48–63	2.25	1.97–2.57	< 0.0001	1.54	1.07–2.23	0.022
≥ 64	7.33	6.45–8.34	< 0.0001	2.26	1.48–3.45	0.0002
Sex (male/female)	1.24	0.96–1.61	0.094			
Smoking						
Non-smoker	Reference			Reference		
Smoker	1.56	1.09–2.22	0.015	1.45	0.95–2.20	0.085
Ex-smoker	1.77	1.19–2.63	0.005	1.36	0.84–2.22	0.206
Active TB	1.73	1.07–2.79	0.024	1.26	0.72–2.21	0.421
History of TB	1.63	0.92–2.88	0.094			
NTM infection	2.00	0.73–5.51	0.181			
Hematologic malignancy	8.21	4.98–13.52	< 0.0001	1.71	0.92–3.16	0.089
Renal insufficiency	3.80	2.16–6.68	< 0.0001	1.02	0.51–2.07	0.948
Solid malignancy	3.91	2.22–6.88	< 0.0001	2.05	1.04–4.01	0.038
Diabetes mellitus	2.95	2.05–4.25	< 0.0001	1.03	0.65–1.63	0.911
Chronic liver disease	6.07	2.54–14.51	< 0.0001	1.92	0.72–5.11	0.192
HIV infection	1.79	0.83–3.87	0.137			
Cardiac disease	2.14	1.45–3.16	0.0001	0.98	0.61–1.57	0.938
COPD	3.20	1.71-6.00	0.0003	1.10	0.52–2.33	0.809
Autoimmune disease	4.59	3.53–5.96	< 0.0001	4.39	3.05–6.32	< 0.0001
Corticosteroids	9.36	7.22–12.13	< 0.0001	4.16	2.99–5.79	< 0.0001
Immunosuppressant	3.58	2.70–4.76	< 0.0001	1.18	0.80–1.74	0.412
Acute Infection	7.16	5.09–10.08	< 0.0001	2.08	1.30–3.31	0.002
Lymphopenia	11.28	8.57–14.85	< 0.0001	3.09	2.14–4.47	< 0.0001
Neutropenia	0.68	0.47–0.99	0.043	0.85	0.54–1.33	0.469
CRP	1.10	1.08–1.12	< 0.0001	1.07	1.05–1.10	< 0.0001
Hypoalbuminemia	15.53	11.87–20.32	< 0.0001	3.06	2.11–4.42	< 0.0001

Cases were divided according to the criterion for indeterminate results (low Mitogen IFN-γ level in QFT-GIT, i.e. Mitogen − Nil IFN-γ level < 0.5 IU/mL). For definitions of lymphopenia, neutropenia, and hypoalbuminemia, refer to the Methods

*Mitogen* mitogen tube, *IFN-γ* interferon-γ, *IGRA* interferon-γ release assay, *QFT-GIT* QuantiFERON-TB Gold-in-Tube, *OR* odds ratio, *CI* confidence interval, *TB* tuberculosis, *NTM* non-tuberculous mycobacteria, *HIV* human immunodeficiency virus, *COPD* chronic obstructive pulmonary disease, *CRP* C-reactive protein, *Nil* nil tube

### Use of TBAg IFN-γ levels to interpret IGRA results

Based on the median value of the TBAg IFN-γ levels of the cases that met the original IGRA cutoff (TBAg – Nil IFN-γ level of 0.35 IU/mL), we arbitrarily set the cutoff IFN-γ level for TBAg to 0.48 IU/mL. As a result, 14/36 cases with IGRA-negative active TB (38.9%) became positive, and 1/376 IGRA-positive active TB cases (0.003%) became negative. Among the non-TB subjects, 284/7202 IGRA-negative cases (3.9%) became positive and 42/1513 IGRA-positive cases (2.8%) became negative. With regard to the indeterminate results, 15/19 cases with IGRA-indeterminate active TB (78.9%) were considered positive using the new cutoff IFN-γ level of TBAg, excluding the Mitogen criterion. Therefore, reinterpretation with the new cutoff TBAg IFN-γ value improved overall sensitivity for the detection of active TB from 87.2 to 93.7%. The specificity for active TB slightly decreased from 80.5 to 79.9%, although the true specificity could not be determined due to the existence of LTBI. The sensitivity and specificity changes could not be assessed comprehensively because conversion from “true” to “false” could not be determined in the non-TB groups due to the inability to diagnose LTBI.

## Discussion

We reinterpreted IGRA results by assessing the implications of the values for individual QFT-GIT kit tubes. Here, we propose a revised IGRA interpretation. First, measurement of the Nil level may be unnecessary, or even detrimental; interpretation based on the TBAg IFN-γ level is preferable. To use this strategy, identification of appropriate cutoff values and ranges is necessary. The TBAg IFN-γ level may be useful, even in cases with a low Mitogen value. A result should not be dismissed as indeterminate merely because the Mitogen level is suboptimal. Due to the variability in the IGRA, its results should be interpreted using a borderline range instead of a point estimate, and clinical interpretation based on risk assessment for TB exposure should be performed for borderline results.

The advantages of IGRA reinterpretation using TBAg IFN-γ levels were demonstrated for the diagnosis of active TB, then reinforced by other analyses including intergroup comparison and logistic regression. We suppose that that the advantages would be further enhanced if all TB infections (including true LTBI) could be analyzed in comparison to the TB-naïve group. Additionally, actual changes in positivity according to the cutoff value are important and not fully reflected in the AUC differences. These changes were documented in our results, although the analyses were also limited to the active TB group. Also, stable and definitive cutoff values could not be defined, particularly due to the presence of abundant LTBI cases, which could not be accurately determined. Further studies in well-defined populations are needed to address this drawback.

Previously, we reported the diagnostic utility of TB-antigen-stimulated IFN-γ-inducible chemokine levels [9]. In TB-antigen-stimulated whole-blood assays, the Nil level is usually subtracted from TBAg values, which may reduce significance. Therefore, we suggested not subtracting the Nil level; however, this concept may not have been widely adopted because it is somewhat counterintuitive.

This study showed that TB infection affects Nil values. First, Nil IFN-γ levels were significantly higher in the active TB group than in the non-TB group; they were also higher in the IGRA-positive non-TB group than in the IGRA-negative non-TB group. Second, Nil and TBAg IFN-γ levels were significantly correlated. Third, the predictive performance of TBAg IFN-γ levels was superior to those of TBAg − Nil in ROC analysis. Fourth, regression analysis showed that the factor with the greatest influence on Nil IFN-γ levels was active TB, followed by previous TB infection. The odds ratio of active TB was greater for a high TBAg IFN-γ level than for a high TBAg − Nil IFN-γ level. These issues have already been discussed [9]. The mechanisms of action of IFN-γ and IFN-γ-inducible chemokines are closely related; the Nil values of IFN-γ are reportedly increased in active TB, but to a lesser degree than the Nil values of IFN-γ-inducible chemokines due to lower concentrations [9]. In the present study, increased Nil IFN-γ levels in active TB were more clearly identified in a large population. Increased Nil IFN-γ levels in active TB have also been reported in other studies [24, 25].

Activated peripheral blood T lymphocytes produce IFN-γ in TB infection [26–28]. This may explain the greater IFN-γ production by lymphocytes in TB-infected subjects than in TB-naïve subjects without TB-antigen stimulation. IFN-γ and CXR3 ligands in peripheral blood are increased without incubation or stimulation, although IFN-γ may be more difficult to analyze relative to other chemokines due to its relatively low concentrations [29–31].

With regard to indeterminate results, our data confirmed the influence of the factors proposed in previous studies [32–36]. In the present study, the use of well-controlled procedures may have contributed to the low incidence of indeterminate results, although the rates of indeterminate results are reportedly high in Asian populations [37]. Notably, acute infection and increased CRP levels were identified as independent contributors to indeterminate results. This may be related to the role of inhibitory receptors in lymphocyte activation during acute infection [38]. The rates of indeterminate results were reportedly higher in patients with COVID-19 [39].

The IGRA-indeterminate non-TB group exhibited a higher lymphocyte and a lower neutrophil count compared to the other groups. In the multivariate analysis, the lymphocyte count remained a significant factor influencing the indeterminate results. This finding is plausible in that the lymphocytes are the main player in the IGRA process. However, the significance of the neutrophil count was lost in the multivariate analysis, possibly due to its association with secondary phenomena caused by infection and other underlying conditions.

Among cases with indeterminate results, many active TB cases had high TBAg IFN-γ levels despite the presence of low Mitogen IFN-γ levels. If the TBAg IFN-γ level is disproportionately high compared to that of Mitogen, this could be used to evaluate positivity. In some cases, low Mitogen IFN-γ levels may result from local issues in the mitogen tube and may not be due to suppressed immunity. Therefore, individual cases should be carefully reviewed. Also, in cases with low Mitogen IFN-γ levels due to the decreased immunity, the possibility of false negative results should be considered, particularly near the cutoff value.

In this study, another finding associated with the variability of the results was that the TBAg − Nil calculations yielded high rates of negative values (> 50% for IGRA-negative non-TB subjects). Typically, the TBAg level is greater than or equal to the Nil level.

TB subjects had significantly higher TBAg IFN-γ levels compared to IGRA-positive subjects. This contradicts the assumption that IFN-γ levels are indistinguishable between active TB and LTBI. Although there was little difference in discriminatory power and a sizable overlap between the two groups, values near the cutoff could be affected. Furthermore, TBAg IFN-γ levels were underestimated in the active TB group: subjects with IFN-γ levels > 10 IU/mL were more prevalent in the active TB group, and IGRA-negative subjects were included in the active TB group but not in the IGRA-positive non-TB group.

Age was linked to high Nil and TBAg IFN-γ levels, as expected, given the decreasing incidence of TB via national control efforts [22]. Additionally, logistic regression analysis showed a higher incidence in men. Several significant predictors in univariate analyses lost significance in age-adjusted multivariate analysis.

The adoption of our interpretation strategy will require validation studies to determine the diagnostic performance in the most relevant populations and settings, including the determination of reference ranges and cut-offs, as these may differ in different population groups and settings.

This study had both limitations and strengths. First, since we included IGRA results from all age groups and clinical settings, there was considerable heterogeneity in the study population. However, the unselective inclusion of the IGRA results of a relatively large population may yield real-world results. Second, validity was reduced by the single-center design. However, because South Korea is a TB-endemic country, our population included active TB, LTBI, and younger TB-naïve subjects. Third, there were missing data for smoking and laboratory parameters because of the retrospective nature of the analysis. The large number of cases may overcome this shortcoming.

In conclusion, the results of our comprehensive assessment and reinterpretation strategy can facilitate tuberculosis control. Our results are a useful addition to the literature on the role of IGRA in the diagnosis of TB infection. Specifically, we suggest the use of TBAg IFN-γ levels for IGRA interpretation, because the use of Nil values may decrease the validity of the results. Mitogen levels may affect, but not absolutely determine, the validity of the results, because TBAg IFN-γ levels can be informative even in cases with low Mitogen IFN-γ levels.

## Electronic supplementary material

Below is the link to the electronic supplementary material.

Additional file 1: table S1. Univariate and multivariate binary logistic regression analyses to identify factors associated with high TBAg IFN-γ levels in the IGRA (QFT-GIT).

Additional file 2: table S2. Univariate and multivariate binary logistic regression analyses to determine factors associated with high TBAg − Nil IFN-γ levels in the IGRA (QFT-GIT).

Additional file 3: table S3. Univariate and multivariate binary logistic regression analyses to identify factors associated with IGRA (QFT-GIT) positivity.

Additional file 4: table S4. Comparison of Nil IFN-γ levels according to the presence of chronic disease.

Additional file 5: table S5. Univariate and multivariate binary logistic regression analyses to identify factors associated with high Nil IFN-γ levels in the IGRA (QFT-GIT), with chronic disease collectively serving as an independent variable.

Additional file 6: table S6. Characteristics of the cases with indeterminate results of IGRA (QFT-GIT).

Additional file 7: figure S1. Forest plot of the significant factors in the multivariate logistic regression to predict high Nil IFN-γ levels in the IGRA (QFT-GIT) with chronic disease collectively serving as an independent variable. *Nil* nil tube, *IFN-γ* interferon-γ, *IGRA* interferon-γ release assay, *QFT-GIT* QuantiFERON-TB Gold-in-Tube.

## Data Availability

The datasets generated during and/or analyzed during the current study are not publicly available due to the regulations and recommendations of the Institutional Review Board, but may be available from the corresponding author on reasonable request after obtaining the approval of the Institutional Review Board.
