# Supplementary material for: Enhancing the interferon-γ release assay through omission of nil and mitogen values
Source: Respir Res. 2023 Jul 7;24:179. doi: 10.1186/s12931-023-02485-4 (PMC10327336; doi:10.1186/s12931-023-02485-4)
Supplement: Supplementary file 1 — Additional file 1: table S1. Univariate and multivariate binary logistic regression analyses to identify factors associated with high TBAg IFN-γ levels in the IGRA (QFT-GIT). [file 12931_2023_2485_MOESM1_ESM.docx]

**Table S1** Univariate and multivariate binary logistic regression analyses to identify factors associated with high TBAg IFN-γ levels in the IGRA (QFT-GIT)

| Variable | Univariate | | | Multivariate | | |
| --- | --- | --- | --- | --- | --- | --- |
|  | OR | 95% CI | *P* value | OR | 95% CI | *P* value |
| Age, years |  | | | | | |
| ≤ 14 | 1.06 | 0.78–1.45 | 0.712 | 0.96 | 0.69–1.34 | 0.836 |
| 15–47 | Reference | | | Reference | | |
| 48–63 | 2.50 | 2.24–2.79 | <0.0001 | 2.20 | 1.95–2.48 | <0.0001 |
| ≥ 64 | 3.45 | 2.89–4.13 | <0.0001 | 2.69 | 2.17–3.32 | <0.0001 |
| Sex (male/female) | 1.29 | 1.19–1.41 | <0.0001 | 1.11 | 1.01–1.22 | 0.038 |
| Smoking status |  | | | | | |
| Non-smoker | Reference | | | Reference | | |
| Smoker | 1.68 | 1.41–2.00 | <0.0001 | 1.07 | 0.87–1.31 | 0.508 |
| Ex-smoker | 1.72 | 1.40–2.12 | <0.0001 | 1.16 | 0.94–1.42 | 0.159 |
| Active TB | 72.52 | 34.32–153.22 | <0.0001 | 62.34 | 27.74–140.08 | <0.0001 |
| History of TB | 7.34 | 5.25–10.27 | <0.0001 | 4.78 | 3.38–6.77 | <0.0001 |
| Recent contact with TB | 0.85 | 0.66–1.11 | 0.227 |  |  |  |
| NTM infection | 2.56 | 1.55–4.22 | 0.0002 | 1.23 | 0.72–2.10 | 0.461 |
| Hematologic malignancy | 1.98 | 1.30–3.02 | 0.001 | 1.26 | 0.80–1.98 | 0.323 |
| Renal insufficiency | 1.60 | 1.14–2.24 | 0.007 | 0.77 | 0.53–1.13 | 0.189 |
| Solid malignancy | 2.02 | 1.42–2.87 | 0.0001 | 1.04 | 0.71–1.53 | 0.838 |
| Diabetes mellitus | 1.81 | 1.50–2.18 | <0.0001 | 1.02 | 0.83–1.27 | 0.828 |
| Chronic liver disease | 1.77 | 0.91–3.45 | 0.092 |  |  |  |
| HIV infection | 0.87 | 0.63–1.21 | 0.416 |  |  |  |
| Cardiac disease | 1.56 | 1.31–1.86 | <0.0001 | 1.10 | 0.91–1.34 | 0.343 |
| COPD | 1.98 | 1.38–2.85 | 0.0002 | 0.93 | 0.62–1.40 | 0.735 |
| Autoimmune disease | 1.18 | 1.04–1.34 | 0.009 | 1.02 | 0.89–1.16 | 0.813 |
| Corticosteroids | 0.99 | 0.85–1.14 | 0.835 |  |  |  |
| Immunosuppressant | 1.08 | 0.94–1.23 | 0.302 |  |  |  |
| Acute infection | 1.35 | 1.06–1.71 | 0.015 | 1.00 | 0.76–1.31 | 0.990 |
| Lymphopenia | 1.43 | 1.20–1.69 | <0.0001 | 0.97 | 0.85–1.10 | 0.635 |
| Neutropenia | 0.96 | 0.84–1.09 | 0.508 |  |  |  |
| CRP | 1.01 | 1.00–1.03 | 0.149 |  |  |  |
| Hypoalbuminemia | 2.00 | 1.64–2.44 | <0.0001 | 0.94 | 0.74–1.20 | 0.618 |

Cases were divided into high- and low-IFN-γ TBAg groups based on the median value (0.14 IU/mL). Cases with indeterminate results were excluded. For definitions of lymphopenia, neutropenia, and hypoalbuminemia, refer to the Methods.

*TBAg* tuberculosis antigen tube, *IFN-γ* interferon-γ, *IGRA* interferon-γ release assay, *QFT-GIT* QuantiFERON-TB Gold-in-Tube, *OR* odds ratio, *CI* confidence interval, *TB* tuberculosis, *NTM* non-tuberculous mycobacteria, *HIV* human immunodeficiency virus, *COPD* chronic obstructive pulmonary disease, *CRP* C-reactive protein
