# Supplementary material for: Enhancing the interferon-γ release assay through omission of nil and mitogen values
Source: Respir Res. 2023 Jul 7;24:179. doi: 10.1186/s12931-023-02485-4 (PMC10327336; doi:10.1186/s12931-023-02485-4)
Supplement: Supplementary file 2 — Additional file 2: table S2. Univariate and multivariate binary logistic regression analyses to determine factors associated with high TBAg − Nil IFN-γ levels in the IGRA (QFT-GIT). [file 12931_2023_2485_MOESM2_ESM.docx]

**Table S2** Univariate and multivariate binary logistic regression analyses to determine factors associated with high TBAg−Nil IFN-γ levels in the IGRA (QFT-GIT)

| Variable | Univariate | | | Multivariate | | |
| --- | --- | --- | --- | --- | --- | --- |
|  | OR | 95% CI | *P* value | OR | 95% CI | *P* value |
| Age groups |  | | | | | |
| ≤ 14 | 0.79 | 0.57-1.08 | 0.138 | 0.73 | 0.52-1.03 | 0.071 |
| 15-47 | Reference | | | Reference | | |
| 48-63 | 2.99 | 2.67-3.35 | <0.0001 | 2.61 | 2.31-2.94 | <0.0001 |
| ≥ 64 | 4.50 | 3.72-5.44 | <0.0001 | 3.55 | 2.84-4.44 | <0.0001 |
| Sex (male/female) | 1.28 | 1.17-1.39 | <0.0001 | 1.08 | 0.98-1.19 | 0.106 |
| Smoking status |  | | | | | |
| Non-smoker | Reference | | | Reference | | |
| Smoker | 1.89 | 1.58-2.25 | <0.0001 | 1.12 | 0.95-1.32 | 0.169 |
| Ex-smoker | 1.78 | 1.45-2.21 | <0.0001 | 1.15 | 0.89-1.48 | 0.275 |
| Active TB | 49.67 | 25.62-96.28 | <0.0001 | 39.09 | 20.09-76.09 | <0.0001 |
| History of TB | 7.55 | 5.34-10.68 | <0.0001 | 4.55 | 3.78-5.47 | <0.0001 |
| Recent contact with TB | 0.91 | 0.70-1.18 | 0.466 |  |  |  |
| NTM infection | 2.57 | 1.55-4.27 | 0.0003 | 1.05 | 0.60-1.82 | 0.868 |
| Hematologic malignancy | 1.86 | 1.22-2.84 | 0.004 | 1.04 | 0.65-1.64 | 0.882 |
| Renal insufficiency | 1.86 | 1.31-2.63 | 0.0005 | 0.83 | 0.56-1.22 | 0.340 |
| Solid malignancy | 2.58 | 1.78-3.75 | <0.0001 | 1.26 | 0.84-1.89 | 0.269 |
| Diabetes mellitus | 2.14 | 1.77-2.59 | <0.0001 | 1.12 | 0.90-1.40 | 0.293 |
| Chronic liver disease | 1.67 | 0.86-3.24 | 0.132 |  |  |  |
| HIV infection | 0.80 | 0.57-1.11 | 0.179 |  |  |  |
| Cardiac disease | 1.52 | 1.28-1.81 | <0.0001 | 0.98 | 0.80-1.19 | 0.817 |
| COPD | 2.90 | 1.96-4.30 | <0.0001 | 1.34 | 0.87-2.06 | 0.186 |
| Autoimmune disease | 1.42 | 1.26-1.61 | <0.0001 | 1.20 | 1.02-1.40 | 0.025 |
| Corticosteroids | 1.01 | 0.87-1.17 | 0.884 |  |  |  |
| Immunosuppressant | 1.24 | 1.08-1.42 | 0.002 | 1.00 | 0.84-1.19 | 0.975 |
| Acute infection | 1.30 | 1.03-1.66 | 0.030 | 0.95 | 0.72-1.25 | 0.727 |
| Lymphopenia | 1.18 | 0.99-1.39 | 0.061 |  |  |  |
| Neutropenia | 0.82 | 0.72-0.94 | 0.004 | 0.96 | 0.82-1.12 | 0.575 |
| CRP | 1.01 | 1.00-1.02 | 0.070 |  |  |  |
| Hypoalbuminemia | 1.88 | 1.54-2.30 | <0.0001 | 0.81 | 0.63-1.04 | 0.105 |

Cases were divided into high- and low-IFN-γ TBAg−Nil groups according to the median value (0.02 IU/mL). Cases with indeterminate results were excluded. For definitions of lymphopenia, neutropenia, and hypoalbuminemia, refer to the Methods.

*TBAg* tuberculosis antigen tube, *Nil* nil tube, *IFN-γ* interferon-γ, *IGRA* interferon-γ release assay, *QFT-GIT* QuantiFERON-TB Gold-in-Tube, *OR* odds ratio, *CI* confidence interval, *TB* tuberculosis, *NTM* non-tuberculous mycobacteria, *HIV* human immunodeficiency virus, *COPD* chronic obstructive pulmonary disease, *CRP* C-reactive protein
