# Supplementary material for: Enhancing the interferon-γ release assay through omission of nil and mitogen values
Source: Respir Res. 2023 Jul 7;24:179. doi: 10.1186/s12931-023-02485-4 (PMC10327336; doi:10.1186/s12931-023-02485-4)
Supplement: Supplementary file 3 — Additional file 3: table S3. Univariate and multivariate binary logistic regression analyses to identify factors associated with IGRA (QFT-GIT) positivity. [file 12931_2023_2485_MOESM3_ESM.docx]

**Table S3** Univariate and multivariate binary logistic regression analyses to identify factors associated with IGRA positivity (QFT-GIT)

| Variable | Univariate | | | Multivariate | | |
| --- | --- | --- | --- | --- | --- | --- |
|  | OR | 95% CI | *P* value | OR | 95% CI | *P* value |
| Age groups |  | | | | | |
| ≤ 14 | 0.65 | 0.38-1.11 | 0.112 | 0.40 | 0.20-0.77 | 0.006 |
| 15-47 | Reference | | | Reference | | |
| 48-63 | 4.59 | 4.07-5.18 | <0.0001 | 4.27 | 3.73-4.89 | <0.0001 |
| ≥ 64 | 5.57 | 4.69-6.61 | <0.0001 | 4.88 | 3.90-6.11 | <0.0001 |
| Sex (male/female) | 1.79 | 1.62-1.98 | <0.0001 | 1.41 | 1.24-1.61 | <0.0001 |
| Smoking status |  | | | | | |
| Non-smoker | Reference | | | Reference | | |
| Smoker | 2.73 | 2.29-3.25 | <0.0001 | 1.42 | 1.12-1.78 | 0.004 |
| Ex-smoker | 2.49 | 2.03-3.06 | <0.0001 | 1.36 | 1.04-1.77 | 0.025 |
| Active TB | 49.72 | 35.16-70.30 | <0.0001 | 46.98 | 32.58-67.73 | <0.0001 |
| History of TB | 9.56 | 7.44-12.29 | <0.0001 | 6.40 | 4.81-8.49 | <0.0001 |
| Recent contact with TB | 1.08 | 0.79-1.47 | 0.632 |  |  |  |
| NTM infection | 1.74 | 1.06-2.85 | 0.029 | 0.48 | 0.26-0.88 | 0.017 |
| Hematologic malignancy | 1.29 | 0.81-2.05 | 0.285 |  |  |  |
| Renal insufficiency | 2.35 | 1.66-3.31 | <0.0001 | 0.93 | 0.60-1.42 | 0.728 |
| Solid malignancy | 2.78 | 1.97-3.93 | <0.0001 | 1.17 | 0.78-1.77 | 0.443 |
| Diabetes mellitus | 2.37 | 1.96-2.86 | <0.0001 | 1.00 | 0.79-1.27 | 0.981 |
| Chronic liver disease | 1.63 | 0.80-3.29 | 0.178 |  |  |  |
| HIV infection | 0.84 | 0.55-1.28 | 0.408 |  |  |  |
| Cardiac disease | 1.70 | 1.41-2.06 | <0.0001 | 0.89 | 0.70-1.14 | 0.361 |
| COPD | 2.60 | 1.82-3.70 | <0.0001 | 0.91 | 0.59-1.40 | 0.655 |
| Autoimmune disease | 1.31 | 1.14-1.52 | <0.0001 | 1.05 | 0.89-1.24 | 0.545 |
| Corticosteroids | 0.96 | 0.80-1.15 | 0.626 |  |  |  |
| Immunosuppressant | 1.12 | 0.95-1.32 | 0.169 |  |  |  |
| Acute infection | 1.31 | 1.00-1.73 | 0.051 |  |  |  |
| Lymphopenia | 1.04 | 0.86-1.26 | 0.663 |  |  |  |
| Neutropenia | 0.72 | 0.61-0.85 | <0.0001 | 0.94 | 0.80-1.11 | 0.456 |
| CRP | 1.00 | 0.98-1.01 | 0.521 |  |  |  |
| Hypoalbuminemia | 2.04 | 1.66-2.50 | <0.0001 | 0.57 | 0.43-0.76 | 0.0001 |

Cases were divided into two groups according to QFT-GIT positivity. Cases with indeterminate results were excluded. For definitions of lymphopenia, neutropenia, and hypoalbuminemia, refer to the Methods.

*IGRA* interferon-γ release assay, *QFT-GIT* QuantiFERON-TB Gold-in-Tube, *OR* odds ratio, *CI* confidence interval, *TB* tuberculosis, *NTM* non-tuberculous mycobacteria, *HIV* human immunodeficiency virus, *COPD* chronic obstructive pulmonary disease, *CRP* C-reactive protein
