# Supplementary material for: Enhancing the interferon-γ release assay through omission of nil and mitogen values
Source: Respir Res. 2023 Jul 7;24:179. doi: 10.1186/s12931-023-02485-4 (PMC10327336; doi:10.1186/s12931-023-02485-4)
Supplement: Supplementary file 4 — Additional file 4: table S4. Comparison of Nil IFN-γ levels according to the presence of chronic disease. [file 12931_2023_2485_MOESM4_ESM.docx]

**Table S4** Comparison of Nil IFN-γ levels according to the presence of chronic disease.

|  | No chronic disease | Chronic disease | P value |
| --- | --- | --- | --- |
| All cases |  |  |  |
| No. of cases | 6,708 | 2,670 |  |
| Nil IFN-γ levels (IU/mL) | 0.09 (0.06-0.17) | 0.08 (0.05-0.16) | 0.410 |
| Cases with active TB |  |  |  |
| No. of cases | 306 | 125 |  |
| Nil IFN-γ levels (IU/mL) | 0.18 (0.09-0.45) | 0.20 (0.10-0.45) | 0.404 |
| Cases with non-TB |  |  |  |
| No. of cases | 6,402 | 2,545 |  |
| Nil IFN-γ levels (IU/mL) | 0.09 (0.06-0.16) | 0.08 (0.05-0.15) | 0.627 |

Data are medians (interquartile range)

*Nil* nil tube, *IFN-γ* interferon-γ, *TB* tuberculosis, *non-TB* non-tuberculosis
