# Supplementary material for: Enhancing the interferon-γ release assay through omission of nil and mitogen values
Source: Respir Res. 2023 Jul 7;24:179. doi: 10.1186/s12931-023-02485-4 (PMC10327336; doi:10.1186/s12931-023-02485-4)
Supplement: Supplementary file 5 — Additional file 5: table S5. Univariate and multivariate binary logistic regression analyses to identify factors associated with high Nil IFN-γ levels in the IGRA (QFT-GIT), with chronic disease collectively serving as an independent variable. [file 12931_2023_2485_MOESM5_ESM.docx]

**Table S5** Univariate and multivariate binary logistic regression analyses to identify factors associated with high Nil IFN-γ levels in the IGRA (QFT-GIT) with chronic diseases collectively as an independent variable

| Variable | Univariate | | | Multivariate | | |
| --- | --- | --- | --- | --- | --- | --- |
|  | OR | 95% CI | *P* value | OR | 95% CI | *P* value |
| Age, years |  | | | | | |
| ≤ 14 | 1.23 | 0.91-1.68 | 0.183 | 1.28 | 0.93-1.76 | 0.131 |
| 15-47 | Reference | | | Reference | | |
| 48-63 | 1.07 | 0.96-1.19 | 0.210 | 1.07 | 0.96-1.20 | 0.237 |
| ≥ 64 | 1.18 | 1.00-1.39 | 0.049 | 1.10 | 0.92-1.32 | 0.280 |
| Sex (male/female) | 0.97 | 0.89-1.05 | 0.432 |  |  |  |
| Smoking status |  | | | | | |
| Non-smoker | Reference | | |  | | |
| Smoker | 0.88 | 0.75-1.05 | 0.147 |  |  |  |
| Ex-smoker | 0.92 | 0.76-1.13 | 0.439 |  |  |  |
| Active TB | 2.87 | 2.31-3.57 | <0.0001 | 2.62 | 2.09-3.27 | <0.0001 |
| History of TB | 1.43 | 1.14-1.81 | 0.002 | 1.28 | 1.00-1.63 | 0.048 |
| Recent contact with TB | 0.75 | 0.58-0.97 | 0.031 | 0.84 | 0.61-1.14 | 0.255 |
| Chronic diseases | 0.88 | 0.80-0.96 | 0.003 | 0.94 | 0.84-1.06 | 0.327 |
| Corticosteroids | 0.89 | 0.77-1.03 | 0.106 |  |  |  |
| Immunosuppressant | 0.75 | 0.65-0.86 | <0.0001 | 0.91 | 0.77-1.06 | 0.288 |
| Acute infection | 0.89 | 0.70-1.13 | 0.329 |  |  |  |
| Lymphopenia | 1.17 | 1.11-1.23 | 0.051 |  |  |  |
| Neutropenia | 1.10 | 0.94-1.29 | 0.224 |  |  |  |
| CRP | 1.01 | 0.99-1.03 | 0.245 |  |  |  |
| Hypoalbuminemia | 1.19 | 0.98-1.44 | 0.081 |  |  |  |

Cases were divided into high- and low-IFN-γ Nil groups based on the median value (0.09 IU/mL). Cases with indeterminate results were excluded. For definitions of lymphopenia, neutropenia, and hypoalbuminemia, refer to the Methods.

*Nil* nil tube, *IFN-γ* interferon-γ, *IGRA* interferon-γ release assay, *QFT-GIT* QuantiFERON-TB Gold-in-Tube, *OR* odds ratio, *CI* confidence interval, *TB* tuberculosis, *CRP* C-reactive protein
