# Supplementary material for: Enhancing the interferon-γ release assay through omission of nil and mitogen values
Source: Respir Res. 2023 Jul 7;24:179. doi: 10.1186/s12931-023-02485-4 (PMC10327336; doi:10.1186/s12931-023-02485-4)
Supplement: Supplementary file 6 — Additional file 6: table S6. Characteristics of the cases with indeterminate results of IGRA (QFT-GIT). [file 12931_2023_2485_MOESM6_ESM.docx]

**Table S6** Characteristics of the cases with indeterminate results of IGRA (QFT-GIT)

|  | Active TB | Non-TB |
| --- | --- | --- |
| No. of cases | 19 | 232 |
| Age, years | 67 (58-81) | 46 (30–64)^a^ |
| Age group |  |  |
| ≤ 14 | 0 (0) | 19 (8.2) |
| 15–47 | 4 (21.1) | 97 (41.8) |
| 48–63 | 3 (15.8) | 57 (24.6) |
| ≥ 64 | 12 (63.2) | 59 (25.4) |
| Male, *n* (%) | 11 (58) | 86 (40.0) |
| Smoking status (*n* = 233) | 18 | 215 |
| Current | 6 (31.6) | 34 (15.8) |
| Ex-smoker | 2 (10.5) | 29 (13.5) |
| Close contact to active TB | 0 (0) | 1 (0.5) |
| History of TB | 2 (10.5) | 10 (4.7) |
| NTM infection | 0 (0) | 3 (1.4) |
| Corticosteroid use | 4 (21.1) | 105 (48.8) |
| Other immunosuppressants | 1 (5.3) | 58 (27.0) |
| Underlying conditions |  |  |
| Diabetes mellitus | 4 (21.1) | 29 (13.5) |
| Autoimmune disease | 1 (5.3) | 86 (40.0) |
| Hematologic malignancy | 1 (5.3) | 19 (8.8) |
| Solid malignancy | 1 (5.3) | 13 (6.0) |
| HIV infection | 8 (1.9) | 7 (3.3) |
| Renal insufficiency | 2 (10.5) | 12 (5.6) |
| Chronic liver disease | 0 (0) | 6 (2.8) |
| COPD | 1 (5.3) | 9 (4.2) |
| Cardiac disease | 1 (5.3) | 27 (12.6) |
| Acute infection | 9 (2.1) | 46 (21.4) |
| Laboratory results |  |  |
| Leukocyte (×10^3^/mm^3^) | 6.7 (5.3–12.9) | 8.8 (5.4–13.8) |
| Lymphocyte (×10^3^/mm^3^) | 0.8 (0.5–1.3) | 0.9 (0.5–1.3) |
| Neutrophil (×10^3^/mm^3^) | 4.8 (4.1–9.8) | 7.1 (4.0–11.7) |
| Albumin (mg/dL) | 2.7 (2.4–3.3) | 3.3 (2.8–3.8) |
| CRP (mg/dL) | 7.9 (2.1–16.5) | 4.7 (0.9–12.1) |

Data are medians (interquartile range) or *n* (%).

*IGRA* interferon-γ release assay, *QFT-GIT* QuantiFERON-TB Gold-in-Tube, *TB* tuberculosis, *non-TB* non-tuberculosis, *NTM* non-tuberculous mycobacteria, *HIV* human immunodeficiency virus, *COPD* chronic obstructive pulmonary disease, *CRP* C-reactive protein.

^a^*P <* 0.0001 vs. Active TB group
