# Supplementary figures and images for: Enhancing the interferon-γ release assay through omission of nil and mitogen values
Source: Respir Res. 2023 Jul 7;24:179. doi: 10.1186/s12931-023-02485-4 (PMC10327336; doi:10.1186/s12931-023-02485-4)

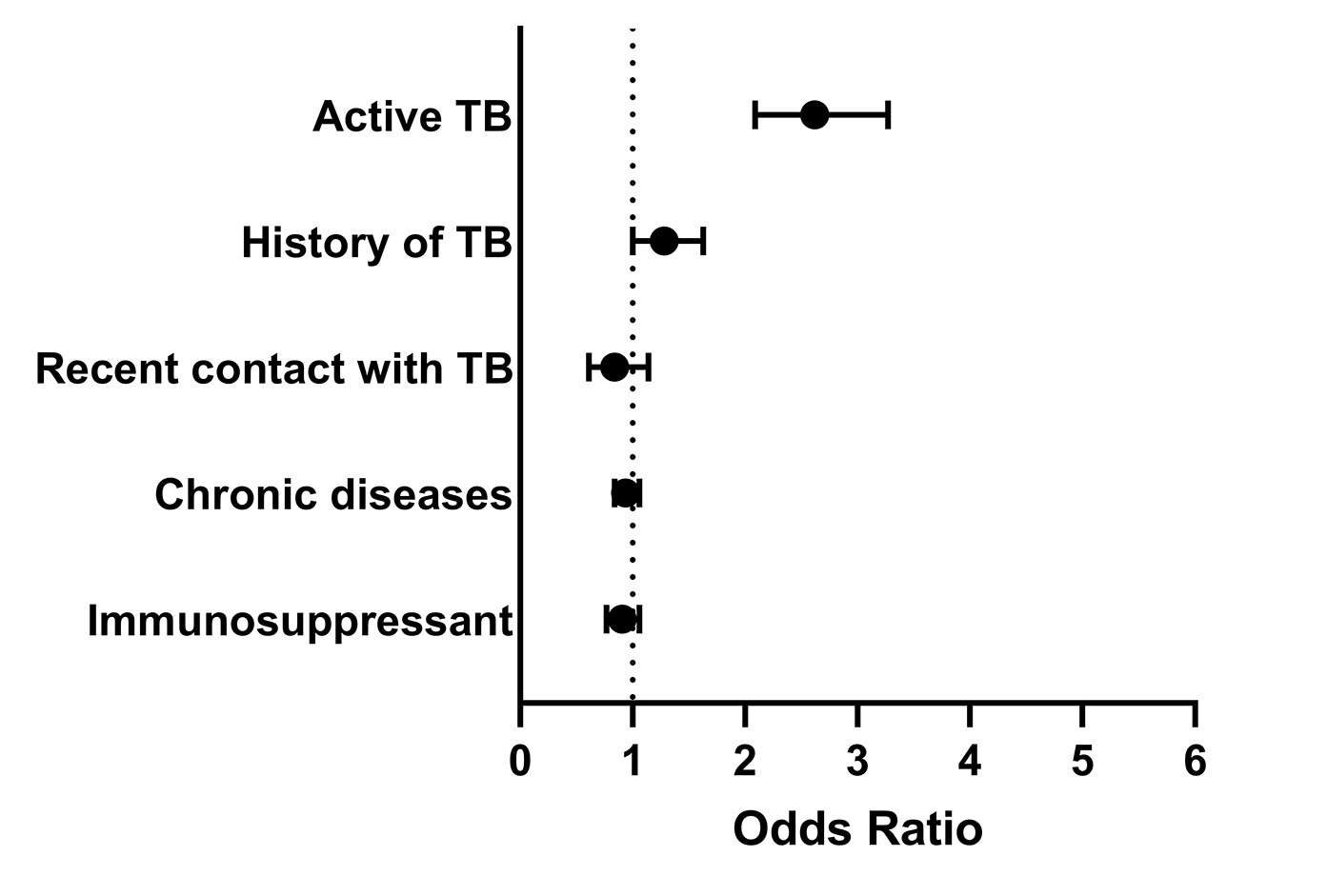

Supplement: Supplementary file 7 — Additional file 7: figure S1. Forest plot of the significant factors in the multivariate logistic regression to predict high Nil IFN-γ levels in the IGRA (QFT-GIT) with chronic disease collectively serving as an independent variable. Nil nil tube, IFN-γ interferon-γ, IGRA interferon-γ release assay, QFT-GIT QuantiFERON-TB Gold-in-Tube. [file 12931_2023_2485_MOESM7_ESM.jpg]
